# Insulin Autoimmune Syndrome: A Case Report Highlighting Diagnostic Pitfalls

**DOI:** 10.7759/cureus.64130

**Published:** 2024-07-09

**Authors:** Hisatoshi Okumura, Satoshi Inaba, Atsushi Kawashima, Taro Shimizu

**Affiliations:** 1 Department of Emergency Medicine, Fukuchiyama City Hospital, Fukuchiyama, JPN; 2 Department of General Medicine, Fukuchiyama City Hospital, Fukuchiyama, JPN; 3 Department of Diagnostic and Generalist Medicine, Dokkyo Medical University Hospital, Mibu, JPN

**Keywords:** non-diabetic adults, hyperinsulinemic hypoglycemia, spontaneous hypoglycemia, exogenous hyperinsulinemia, insulin autoimmune syndrome

## Abstract

Insulin autoimmune syndrome (IAS) is characterized by spontaneous hyperinsulinemic hypoglycemia and the presence of insulin autoantibodies in high titers without exogenous insulin use. The C-peptide level during a hypoglycemia episode is useful for differentiating spontaneous hypoglycemia. Generally, low C-peptides are suspicious for exogenous insulin administration. We report a 47-year-old male nurse who presented with an initial episode of hypoglycemia. Despite the pattern of hyperinsulinemic hypoglycemia and low C-peptide, he was diagnosed with IAS based on the presence of insulin autoantibodies. This case underscores the importance of suspecting IAS in non-diabetic adults with hypoglycemia, even in the setting of low C-peptide levels.

## Introduction

Insulin autoimmune syndrome (IAS), also known as Hirata’s disease, is a rare cause of hyperinsulinemic hypoglycemia, which was first described by Hirata et al. in 1970 [1]. It is characterized by hyperinsulinemic hypoglycemia, elevated insulin autoantibody (IAA) titers, no prior exposure to exogenous insulin, and no pathological abnormalities of pancreatic islets. IAS occurs more frequently in patients over the age of 40 [2]. IAS is more common in East-Asian countries than in Western countries [2]. IAS is the second most common cause of hypoglycemia in Japan, although it is rare [3]. The most recent Japanese survey on the epidemiology of endogenous hyperinsulinemic hypoglycemia reported that an estimated prevalence of IAS was 0.017 cases per 100,000 in the general population [4]. Underdiagnosis is common, particularly in Western countries, and the number of cases among White people has increased [2].

The C-peptide level is useful for differentiating between endogenous and exogenous forms of hyperinsulinemic hypoglycemia. In general, low C-peptide is suspicious for exogenous insulin administration.

We herein report a previously healthy 47-year-old male healthcare provider who presented with an initial episode of hypoglycemia. He was diagnosed with IAS based on high serum concentrations of insulin autoantibodies, despite the pattern of hyperinsulinaemic hypoglycemia and low C-peptide. This case highlights two diagnostic pitfalls in diagnosing spontaneous hypoglycemia in adults: First, he was a healthcare provider, which raised the possibility that he could have likely taken exogenous insulin. Second, he showed low C-peptide, which should be also considered for exogenous insulin administration.

## Case presentation

A previously healthy Japanese 47-year-old male healthcare provider presented with an initial episode of hypoglycemia. Sixteen hours prior to the presentation, he had his usual dinner around 7 pm. However, at 4 am in the morning, he woke up with dizziness, visual disturbance, and sweating. He consumed a banana believing these symptoms arose from hypoglycemia, but his symptoms did not improve overnight. Despite the patient persevering through their work while enduring the symptoms, his symptoms worsened. Therefore, he visited the general internal medicine department at 11 am. He denied fever, sore throat, and rhinorrhea. He had never experienced similar symptoms previously. He had no personal medical history, but his mother had gastric carcinoma. There was no family history of autoimmune disease or hematological disease. He was not taking any medication, including supplements. He had only ever used acetaminophen. He also had never used the exogenous insulin previously. He neither smoked nor consumed alcohol. 

On examination, the temperature was 35.8 °C, blood pressure was 126/78 mmHg, pulse was 78 beats per minute, respiratory rate was 16 breaths per minute, and oxygen saturation was 99% while breathing ambient air. The Glasgow Coma Scale score was 15 (E4, V5, M6). His height was 179.5 cm, and his body weight was 65.5 kg, with a BMI of 20.4. His pupils were equal and reactive to light. There were no motor or sensory deficits. There was no dysmetria or dysdiadochokinesia. The rest of the examinations, including pulmonary, cardiovascular, and abdominal examinations, were normal.

Ambulatory fingerstick glucose was 33 mg/dL. Immediate correction of hypoglycemia was performed using 50% glucose 40 mL, resulting in an improvement of all symptoms as blood glucose levels increased.

Laboratory values on his presentation showed elevation of serum insulin and suppression of serum C-peptide (Table 1). The insulin antibody was positive. His human leukocyte antigen (HLA) was HLA-DR4. A 72-hour fast test did not induce hypoglycemia. The antinuclear antibody was negative. An abdominal computed tomography with contrast showed no pancreatic mass or other intraabdominal abnormalities.

**Table 1:** Laboratory values on the patient's presentation showing elevation of serum insulin and suppression of serum C-peptide

Variable	Reference range, adults	On presentation
Leukocyte count (per μL)	3600–9000	17,700
Hemoglobin (g/dL)	11.3-15.2	16.1
Platelet count (per μL)	12.0-34.0 ×10^4^	32.3×10^4^
HbA1c (%)	4.6-6.2	5.8
Blood glucose level (mg/dL)	70-110	33
Aspartate aminotransferase (U/L)	8-38	32
Alanine aminotransferase (U/L)	4-44	28
Lactate dehydrogenase (U/L)	124-222	206
Creatine kinase (U/L)	56-244	317
Creatinine (mg/dL)	0.30-0.80	0.56
C-reactive protein (mg/dL)	< 0.3	0.06
Serum sodium (mEq/L)	136-147	139
Serum potassium (mEq/L)	3.6-5.0	2.8
Serum chloride (mEq/L)	98-109	99
Serum insulin (μIU/mL)	1.84–12.2	86.3
Serum C-peptide (ng/mL)	0.61–2.09	0.1
insulin antibody (U/mL )	<0.4	3201

Although he is a healthcare provider and has access to exogenous insulin, he completely denied the use of exogenous insulin. He was diagnosed with IAS based on the high titer of insulin antibodies without a history of exogenous insulin use and the presence of HLA-DR4.

He was advised to consume frequent and small meals. One year later, he visited our department again due to hypoglycemia (blood sugar 40 mg/dL) accompanied by dizziness and sweating. At that time, IAA was still positive. After consulting with the diabetes department, the patient was provided with a blood glucose meter and monitored without initiating steroid treatment.

## Discussion

We reported the case of a previously healthy 47-year-old male healthcare provider who presented with an initial episode of hypoglycemia and was diagnosed with IAS. Low C-peptide should be considered for exogenous insulin administration. However, if there is a considerable amount of time between the onset of hypoglycemic episodes and the blood test, seven hours in this case, C-peptide levels may be low even in cases of IAS, leading to diagnostic bias against IAS diagnosis. 

Although the pathophysiology of IAS is not fully understood, it is thought that IAS results from the interaction of a genetic predisposition with environmental triggers, leading to the production of IAA [5]. IAS is strongly associated with HLA-DR4 [6]. HLA-DRB1*0406 is the most frequent HLA-type in East Asian patients, whereas HLA-DRB1*0403 and HLA-DRB*0404 are more frequent in non-East Asian patients [7]. The higher prevalence of HLA-DRB1*0406 in Asian populations has been suggested as a determinant of the higher incidence of IAS in Japan compared to Western countries [8]. Many triggers for the development of IAS have been reported, including drugs, viruses, and hematological disorders [5]. Approximately 80% of IAS cases coexist with other autoimmune diseases. Most commonly, patients have Graves' disease, but others may have systemic lupus erythematosus or rheumatoid arthritis [5]. Among the various drugs as triggers that have been reported, methimazole and alpha-lipoic acid are thought to be drugs more commonly associated with the development of IAS [9,10]. Meanwhile, spontaneous-onset IAS, as in this case, has been reported mostly in Japan [11].

The timing of hypoglycemia in IAS is post-prandial. The presumed mechanism of hypoglycemia in IAS is a mismatch between the glucose level and the free insulin concentration, resulting from the formation of insulin-IAA complexes after insulin is released postprandially. Under this mismatch, the insulin is not fully effective, which leads to transient postprandial hyperglycemia. Then, hyperglycemia triggers the production of more insulin, causing profound hyperinsulinemia. When the binding capacity of insulin autoantibodies is exceeded, the glucose level significantly falls [12]. 

Differential diagnoses of IAS include adrenal insufficiency, insulinoma, exogenous insulin administration, oral hypoglycemic agent administration, medications causing hypoglycemia, type B insulin resistance syndrome, nesidioblastosis, beta-cell hypertrophy, and insulin growth factor 2-producing paraneoplastic syndrome (Table 2) [5,12]. Although rare, IAS is one of the causes of hypoglycemia and should be considered in any patient presenting with hypoglycemia [13]. The measurement of the IAA titer is essential for the diagnosis of IAS [14]. IAA should be measured in non-diabetic adults with hyperinsulinemic hypoglycemia. C-peptide levels are useful for differentiating spontaneous hyperinsulinemic hypoglycemia in adults. If the C-peptide level is high or inappropriately normal, either the intake of exogenous hypoglycemic agents, including sulfonylureas, or insulinoma warrants consideration. However, if the C-peptide level is low, exogenous insulin administration should be suspected [2].

**Table 2:** Differentiation of hypoglycemia The C-peptide level is useful for differentiating between endogenous and exogenous forms of hyperinsulinemic hypoglycemia. The measurement of the insulin autoantibody titer is essential for the diagnosis of insulin antibody syndrome.

Cause	Insulin	C-peptide	Insulin Autoantibody
Insulin autoimmune syndrome	↑↑	↑↑	+
Insulinoma	↑	↑	-
Beta-cell hypertrophy	↑	↑	-
Oral hypoglycemic agent administration	↑	↑	-
Exogenous insulin administration	↑↑	↓	±
Type B insulin resistance syndrome	↑↑	↑↑	-
Nesidioblastosis	↑	↑	-
Insulin growth factor 2 producing paraneoplastic syndrome	↓	↓	-

Insulin and C-peptide are co-secreted in an equimolar ratio by pancreatic beta cells into the portal circulation. Insulin is mainly metabolized by the liver, whereas C-peptide is mainly metabolized by the kidneys, which is much slower. Therefore, the half-life of insulin is five to 10 minutes, whereas that of C-peptide is 30-35 minutes [5]. In IAS, the half-life of insulin increases from five minutes to hours because of IAA bound to insulin, while that of C-peptide usually remains unaffected [15]. Thus, the normal ratio of insulin to C-peptide is less than 1 [16]. Generally, there are two situations that result in a ratio of insulin to C-peptide greater than 1 [12]. One situation is IAS, and the other is exogenous insulin administration. In IAS, the insulin level is elevated, while the C-peptide level may or may not be raised, which is partly dependent on the characteristics of the IAA produced and the type of laboratory assay used [2]. In this case, the insulin level was elevated while the C-peptide level was low, suggesting the possibility of exogenous insulin administration as the cause. In addition, the fact that the patient was a healthcare professional, which raised the possibility that he could have taken exogenous insulin, was also a red herring. The patient was diagnosed with IAS because of a negative history of exogenous insulin use with the positive results of IAA and HLA-DR4. Considering that there was a considerable time between the onset of hypoglycemic symptoms and the visit to our clinic, this may have resulted in a high insulin level and a low C-peptide level.

IAS is mainly a spontaneously remitting disease after the withdrawal of the trigger. A study conducted in Japan in 1992, which included 197 cases of IAS, showed that about 82% of patients with IAS experienced spontaneous remission without any specific treatment [17]. Patients are usually advised to consume frequent, small meals low in carbohydrates to avoid post-prandial hypoglycemia. In severe cases, high-dose corticosteroids may be administered [5]. Several case reports have described the cases of IAS that relapsed despite steroid therapy [18]. Other agents for the treatment include azathioprine, rituximab, an anti-CD20 monoclonal antibody, and plasmapheresis [19-20].

## Conclusions

Low C-peptide should be considered for exogenous insulin administration. However, if there is a considerable amount of time between the onset of hypoglycemic episodes and the blood test, C-peptide levels may be low even in cases of IAS. This case underscores the importance of suspecting IAS in non-diabetic adults with hypoglycemia, even in the setting of a low C-peptide level.
